# circGSK3β promotes metastasis in esophageal squamous cell carcinoma by augmenting β-catenin signaling

**DOI:** 10.1186/s12943-019-1095-y

**Published:** 2019-11-14

**Authors:** Xueting Hu, Duoguang Wu, Xiaotian He, Huiying Zhao, Zhanghai He, Jiatong Lin, Kefeng Wang, Wenjian Wang, Zihao Pan, Huayue Lin, Minghui Wang

**Affiliations:** 1 Guangdong Provincial Key Laboratory of Malignant Tumor Epigenetics and Gene Regulation, Sun Yat-sen Memorial Hospital, Sun Yat-sen University, Guangzhou, China; 2 Department of Thoracic Surgery, Sun Yat-sen Memorial Hospital, Sun Yat-sen University, 107 Yanjiang West Road, Guangzhou, 510120 China; 3 Medical Research Center, Sun Yat-sen Memorial Hospital, Sun Yat-sen University, Guangzhou, China; 4 Department of Pathology, Sun Yat-sen Memorial Hospital, Sun Yat-sen University, Guangzhou, China; 5 Breast Tumor Center, Sun Yat-sen Memorial Hospital, Sun Yat-sen University, 107 Yanjiang West Road, Guangzhou, 510120 China

**Keywords:** Esophageal squamous cell carcinoma (ESCC), circGSK3β, Metastasis, Biomarker

## Abstract

**Background:**

Circular RNAs (circRNAs), a novel class of noncoding RNAs, have recently drawn much attention in the pathogenesis of human cancers. However, the role of circRNAs in esophageal squamous cell carcinoma (ESCC) remains unclear. In this study, we aimed to identify novel circRNAs that regulate ESCC progression and explored their regulatory mechanisms and clinical significance in ESCC.

**Methods:**

Differentially expressed circRNAs between ESCC and paired adjacent normal tissues were identified using microarrays. The effects of a specific differentially expressed circRNA (circGSK3β) on tumor progression were explored in vitro and in vivo. Plasma samples from patients with ESCC, benign lesions and healthy controls were subjected to droplet digital PCR (ddPCR) analyses for circGSK3β, and the detection rates of plasma circGSK3β for ESCC were investigated.

**Results:**

We demonstrated that upregulated expression of circGSK3β was positively associated with advanced clinical stage and poor outcome in patients with ESCC. We further revealed that circGSK3β promoted ESCC cell migration and invasion via direct interaction with GSK3β and inhibiting GSK3β activity, providing a novel mechanism of circRNA in cancer progression. Importantly, we identified that circGSK3β expression in plasma was a biomarker for detection of ESCC and early stage of ESCC with the area under curve (AUC) of 0.782 and 0.793, respectively.

**Conclusions:**

CircGSK3β exerts critical roles in promoting ESCC metastasis and may serve as a novel therapeutic target for ESCC patients. The plasma level of circGSK3β have potential to serve as a novel diagnostic and prognostic biomarker for ESCC detection.

## Background

Esophageal carcinoma is a serious malignancy in terms of both mortality and prognosis [1]. Esophageal squamous cell carcinoma (ESCC) is the most common type of esophageal cancers and accounts for 90% of all cases. Due to recurrence, extensive invasion and metastasis, the overall 5-year survival rate of ESCC is lower than 13% after initial diagnosis [2, 3]. Although multiple gene mutations, including TP53, PIK3CA, EGFR, and KRAS, have been extensively reported in ESCC [4, 5], comprehensive molecular mechanisms that underlie the initiation, progression, and metastasis of ESCC remain elusive. Since early detection is critical for improving outcomes and reducing mortality of ESCC patients, it is of utmost urgency to identify novel therapeutic targets and effective diagnostic markers in ESCC.

Circular RNAs (circRNAs) are a class of non-coding RNAs with a covalently closed loop structure that lack 5′-3′ polarity or a polyadenylated tail [3]. With the development of deep RNA sequencing, a large number of exonic and intronic circRNAs have been identified to be expressed in a tissue-specific manner and in pathological conditions [6], indicating that circRNAs are not simply byproducts of splicing errors. Recent research into circRNA biogenesis has begun to reveal that some circRNAs play important roles in cancer development [7–10]. However, the functions and underlying mechanisms of certain dysregulated circRNAs in ESCC progression remain largely unknown.

Unlike the well-established mechanism of microRNA (miRNA), which is based on seed sequence base-pairing [11], circRNAs’ mode of action remains to be fully elucidated. Some circRNAs exert their functions as miRNA sponges through modulating the activity of miRNAs on other target genes, whereas others are reported to act through binding to RNA-associated proteins to regulate gene transcription, and yet some others are reported to be translatable [12–14]. However, it remains unclear whether circRNAs can regulate its parental protein activation by interacting with and post-translationally modifying signaling proteins.

Since early detection is critical for improving outcomes and reducing mortality of ESCC patients, there is an urgent need to identify biomarkers for early diagnosis and prognosis of the disease. Currently, traditional tumor markers, such as carcinoembryonic antigen (CEA) is used to diagnose and evaluate ESCC progression. However, the sensitivity and validity of CEA detection are insufficient for early cancer detection [15]. It has been reported that circRNAs can be released into circulation [16]. Since plasma circRNAs are protected from RNase digestions, they remain stable for a long period of time even under extreme conditions [17]. The stability and easy detectability make circulating circRNAs an ideal candidate to serve as a biomarker for cancer detection.

In this study, by using a circRNA microarray profiling, we identified circGSK3β as one of the critical circRNAs that was frequently upregulated in ESCC tissues. Patients with higher expression of circGSK3β were associated with poorer survival. Further mechanistic studies demonstrated that circGSK3β promoted metastasis of ESCC cells via interaction with GSK3β and protecting β-catenin from phosphorylation and degradation, which represents a different layer of negative regulation on GSK3β/β-Catenin pathway. Furthermore, we revealed, for the first time, that circGSK3β was upregulated in serum from patients with ESCC, and showed high detection rates for early stage of ESCC.

## Methods

### Patient samples

Patient samples including 50 primary human ESCC and 50 corresponding adjacent non-cancerous tissues were collected between 2017 and 2018 from Sun Yat-sen Memorial Hospital. Clinical information of the patients with ESCC was summarized in Table 1. Blood samples were collected from ESCC patients and age- and gender-matched healthy individuals with no history of cancer and in good health conditions. The blood samples were collected from patients before surgery, chemotherapy, or radiotherapy. At 10 days post-operation, paired plasma samples were collected from 20 randomly selected patients. All plasma samples were extracted from EDTA-K2 tubes and centrifuged as described previously [18]. After the first centrifugation for 10 min at 1600 g, the supernatants were carefully removed and transferred to a new tube follow by centrifugation again at 16,000 g for 10 min to remove residual blood cells. The plasma was then divided into small aliquots and snap-frozen at − 80°C.

**Table 1:** Univariate and multivariate analysis for associations between circGSK3β expression and patient features of ESCC

Feature	*n*	Univariate Analysis				Multivariate Analysis			
		OR	95%CI		*P*	OR	95%CI		*P*
Gender									
Male	38	1							
Female	12	1.08	0.19	6.32	0.542				
Age									
< 60	21	1							
≥ 60	29	0.59	0.17	2.10	0.233				
Histologic grade									
Low	20	1							
Middle/High	30	1.50	0.42	5.32	0.128				
Lymph node metastasis									
Absent	18	1				1			
Present	32	19.3	4.14	90.23	0.014^*^	20.0	4.19	95.22	0.010^*^
TNM stage									
I-II	20	1				1			
III-IV	30	13.5	3.04	58.96	0.001^*^	13.3	2.99	59.25	0.172

*n* sample number, *OR* Odd ratio, *CI* Confidence interval, ^*^P < 0.05 is considered significant

### Microarray analysis

The circRNA Expression Microarray (Aksomics) was used to investigate the differentially expressed circRNAs in three pairs of ESCC and adjacent normal tissues. All primary data in the microarray analysis have been uploaded to the Gene Expression Omnibus with the accession number GSE131969.

### Cell culture and reagents

Human ESCC TE1 cells were grown in the RPMI-1640 medium (Gibco), human ESCC KYSE180 cells were grown in the DMEM medium (Gibco), with supplementation of 10% (v/v) fetal bovine serum (Gibco) and 100 units/ml streptomycin and penicillin (Millipore) in a humidified chamber at 37 °C. For the establishment of stable circGSK3β-depleted cell line, TE1 cells were cultured for 24 h to ~ 80% confluence before transfected with the circGSK3β-shRNA constructs in pLKD vector. The pLKD empty vector was used as a negative control. Twenty four hours post-transfection, the cells were selected with 2 mg/ml puromycin for 2 weeks. The efficiency of the depletion was determined by real-time RT-PCR. The anti-GSK3β antibody, anti-GAPDH antibody, anti-E-cadherin antibody, anti-N-cadherin antibody, anti-Vimentin antibody, anti-Claudin antibody, anti-β-Actin antibody, and anti-β-catenin antibody were from Cell Signaling.

### DNA constructs and transfection

Expression vectors carrying the circGSK3β were constructed by sub-cloning the PCR product of human circGSK3β into pcDNA3.1 vector and were confirmed by sequencing. siRNA targeting circGSK3β, β-catenin and the control siRNA were purchased from RiboBio. Transfections of siRNA and plasmid were performed using the Lipofectamine 3000 Transfection Reagent (Thermo Fisher) according to the manufacturer’s protocol.

### Plasma circRNA isolation and detection

circRNA was isolated from 200 μl plasma samples using the TRIzol LS Reagent (Invitrogen) according to the manufacturer’s instructions. λ-poly A RNA (TAKARA) was added to each plasma specimen at a final concentration of 5 nmol/ml as a reference before isolation. The purified circRNAs were dissolved in 30 μl RNase-free water at a concentration ranging from 5 to 50 ng/ μl.

### Droplet digital PCR (ddPCR)

The ddPCR was constructed on the Naica Crystal Digital PCR System (Stilla Technologies) using ddPCR EvaGreen mix (APExBio). The droplets generation procedure was done according to the manufacturer’s protocol. The cycling protocol was as follows: hot-start at 95 °C for 10 min; followed by 45 cycles of 95 °C for 30 s, 54 °C for 15 s, and 72 °C for 30 s. The data was read by Crystal Reader (Stilla Technologies).

### Quantitative real-time RT-PCR

Total RNA was extracted from cells using Trizol (TIANGEN) according to the manufacturer’s protocol and the cDNA was synthesized using the Revert Aid First Strand cDNA Synthesis Kits (Fermentas). Quantitative real-time RT-PCR was performed using gene-specific primers. The expression of target transcripts was normalized to the β-Actin internal control, and relative changes of gene expression were determined using the 2^−ΔΔCt^ method. The primers for circGSK3β are 5′- TCCTGTTCCTGACGAATCCT − 3′ (forward) and 5′- TACACCAACTGCCCGACTAA − 3′ (reverse), β-Actin are 5′- TCGTGCGTGACATTAAGGAG − 3′ (forward) and 5′- GTCAGGCAGCTCGTAGCTCT − 3′ (reverse), GSK3β are 5′- ACTTCTTGTGGCCTGTCTGG − 3′ (forward) and 5′- AGCTTTTGGCAGCATGAAAG − 3′ (reverse), and β-catenin are 5′- CATCTACACAGTTTGATGCTGCT − 3′ (forward) and 5′- GCAGTTTTGTCAGTTCAGGGA − 3′ (reverse).

### Wound healing assay

Cells were seeded in 6-well plates at a density of 3 × 10^5^ cells per well and incubated for 48 h. Then a 20 μl pipette tip was used to scratch a linear wound in the cell monolayer. Photos were taken at 0, 24, 48, and 72 h after the scrapping, respectively.

### Cell migration assay

Chemotactic migration of TE1 cells and KYSE180 cells were examined using the Transwell Chamber Assay. Cells were serum-starved for 24 h and then plated at the density of 10^5^ cells per well in the serum-free medium. The RPMI 1640 medium containing 10% FBS was added into the bottom chamber as a chemoattractant. After incubation for 24 h, non-migrating cells were removed from the upper chamber and cells migrated through the membrane were fixed with 4% formaldehyde and stained with crystal violet staining solutions. Cell numbers in five randomly selected fields were counted under a microscope (Leica).

### Matrigel invasion assay

The Matrigel Invasion chambers with 8 μm Matrigel coated filters (BD) were used to study the cell invasion activity. Briefly, cells were serum starved for 24 h and then seeded in the upper compartment of Matrigel-coated inserts. The medium containing 10% FBS was added into the bottom chamber. After incubation for 24 h, non-invaded cells in the upper chamber were removed. Cells that invaded through the membrane to the lower surface were counted under a microscope (Leica).

### Flow cytometry

Cells were stained with fluorochrome-conjugated monoclonal antibodies against N-cadherin (BioLegend) and E-cadherin (BioLegend) according to the manufacturer’s instructions, and they were subsequently analyzed by multicolor flow cytometry using Cell Quest software (FACS Verse, BD Immunocytometry Systems).

### Immunoprecipitation and immunoblotting

Immunoprecipitation (IP) and immunoblotting (IB) were performed as previously described [19]. Briefly, cell lysates prepared using lysis buffer TNTE 0.5% (50 mM Tris-HCl, 150 mM NaCl, 1 mM EDTA, and 0.5% Triton X-100, containing 10 mg/ml pepstatin A, 10 mg/ml leupeptin, and 1 mM PMSF) were applied to IP or IB with appropriate antibodies. Proteins were detected by Chemiluminescent HRP substrates (Millipore).

### RNA-binding protein Immunoprecipitation (RIP) and RNA pull down

RIP assay and RNA pull down assay were performed using Megna RIP RNA-Binding Protein Immunoprecipitation kit (Millipore) and Magnetic RNA-Protein Pull-Down Kit (Thermo) according to the manufacturer’s protocol, respectively. The RNA probe complementary to circGSK3β was from Sangon. The bead-binding proteins were eluted by boiling in the loading buffer and then subjected to SDS-PAGE. The proteins were detected with silver staining, and specific bands were excised and analyzed by mass spectrometry or Western blot.

### RNA FISH and immunofluorescence assay

RNA fluorescence in situ hybridization (FISH) was performed using specific probe (Sangon) for the back-splice region of circGSK3β sequence. TE1 cells were fixed with 4% paraformaldehyde. After pre-hybridization with 1 × PBS/0.5% Triton X-100, cells were blocked and hybridized in hybridization buffer with Alexa Fluor 594-conjugated probes at 37 °C overnight. GSK3β proteins were detected using anti-GSK3β antibody followed by Alexa Fluor 488-conjugated secondary antibody (Cell Signaling). For nuclear counterstaining, cells were incubated with DAPI (Solarbio) for 5 min. The images were acquired on a Zeiss LSM 710 confocal microscope (Zeiss).

### Tumorigenicity assay in nude mice

TE1 cells with or without depletion of circGSK3β were used for the tumorigenicity assays. Both groups of cells (5 × 10^6^) were suspended in 100 μl PBS were injected subcutaneously into the flank of BALB/c mice. Tumor volume was determined by external measurement according to the formula volume (mm^3^) = width^2^ x length/2 and the growth of tumors were monitored for 30 days. All mice were then sacrificed and tumors were excised, weighed, and fixed with 10% formaldehyde. All mice were kept under specific pathogen-free conditions at Sun Yat-Sen University Laboratory Animal Center (Sun Yat-sen University, China) in accordance with institutional guidelines. All experimental protocols were approved by the Ethics Committee for Animal Experimentation of Sun Yat-Sen University.

### Immunohistochemistry assay

Tissues were fixed, dehydrated, embedded, and sectioned according to standard procedures. Antigens were retrieved by boiling in the 10 mM citrate buffer for 3 min, and then incubated in 3% hydrogen peroxide for 20 min to block endogenous peroxidase. All sections were incubated with primary antibodies at 4°C overnight. The Ultra-Sensitive SP kit (Maxim) was then used to detect the specifically bound primary antibodies.

### Biochemical analyses

The plasma concentration of CEA was measured by using the Roche High-sensitivity Assay kit performed on a Cobas e601 System (Roche). The cut-off point of CEA is 5 ng/ml. Samples were randomized for testing and blinded to the trained clinical laboratory technician who analyzed and interpreted the data.

### Statistical analysis

The data were expressed as mean ± S.D. of triplicate. Comparison of non-normal data was analyzed using Mann-Whitney U test, and analysis of normalized data was performed using the Student’s *t-test* between two groups. Univariate analysis and multivariate logistic regression were performed to analysis the association between patient characteristics and circGSK3β expression. Overall survival (OS) and metastasis-free survival (MFS) were estimated by Kaplan-Meier method. Receiver operating characteristic (ROC) curves were applied to analysis the diagnostic values of circRNA and CEA. Youden index (sensitivity + specificity-1) was chosen to identify the optimal cut-off threshold values. All *P* values were based on two-sided testing and statistical analysis was performed using SPSS 20.0 statistical software. *P*< 0.05 was considered statistically significant.

## Results

### Overexpression of circGSK3β in ESCC tissues

**Fig. 1:**
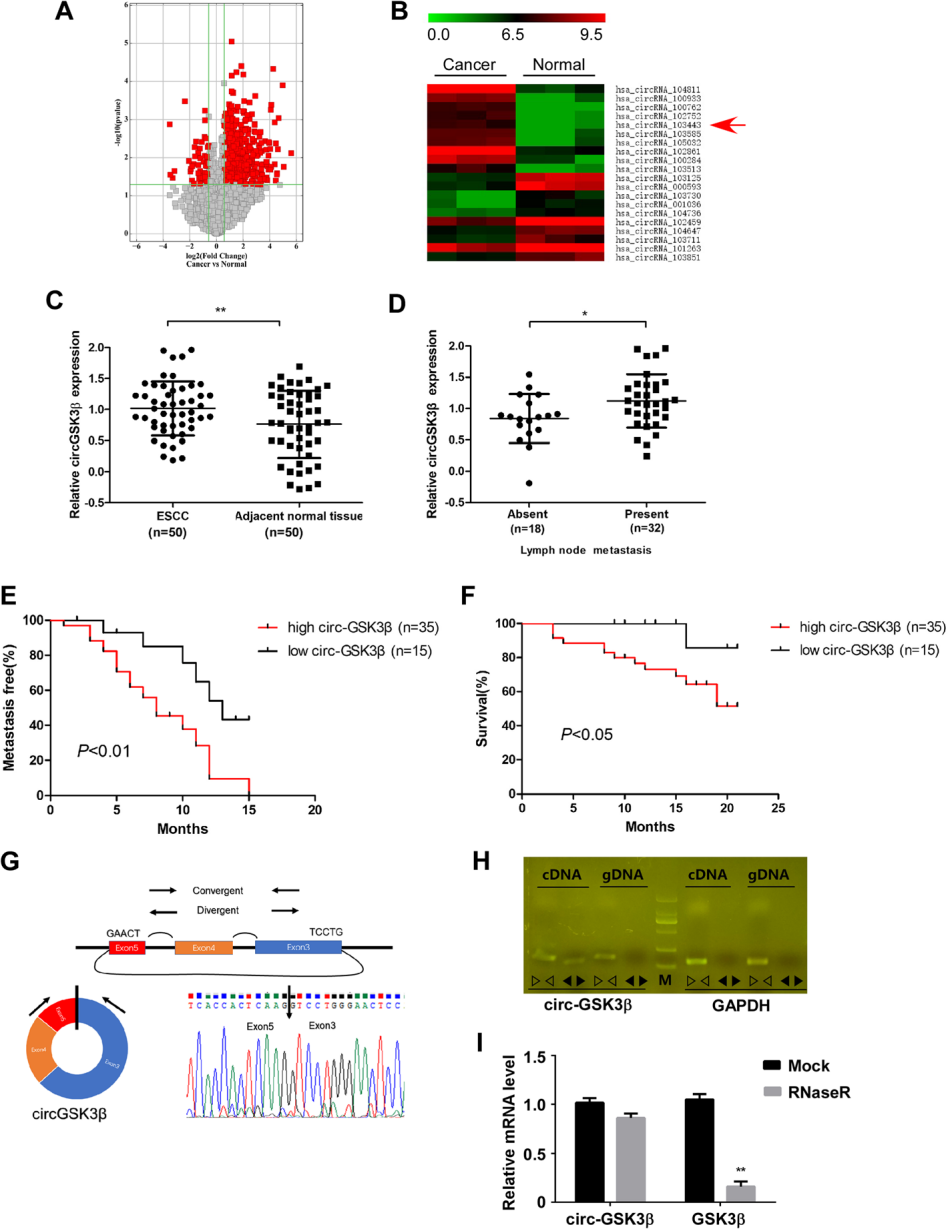
CircGSK3β was overexpressed in ESCC and correlates with poor patient prognosis. **a** Volcano plot compared the expression fold changes of circRNAs for ESCC tissues versus adjacent normal tissues. The red dots represented circRNAs with significantly changed expression level. **b** Clustered heatmap for top 20 upregulated and downregulated circRNAs, with rows representing circRNAs and columns representing tissues. The numerical data represented the serial number of circRNAs in circBase. **c** Scatter plots illustrating qRT-PCR analysis of expression fold change for circGSK3β in ESCC tissues compared with matched adjacent normal tissues. **d** Statistical analyses of circGSK3β expressions in different lymph node metastasis samples. **e** and **f** Statistical analyses of the association of circGSK3β expression with MFS (**e**) and OS (**f**) in ESCC patients. **g** Schematic illustration of circGSK3β locus with specific primers and Sanger sequencing result of circGSK3β. **h** RT-PCR products with divergent primers showing circularization of circGSK3β. **i** RT-qPCR analysis of circGSK3β and GSK3β transcripts in the presence or absence of RNase R treatment, respectively

To investigate the circRNA expression profile in ESCC tissues, we compared three pairs of ESCC tissues and their matched adjacent normal tissues by using circRNA microarray. The variation of circRNA expression was demonstrated in the volcano plot on the basis of the log_2_ (fold changes) ≥1, *p* < 0.05 (Fig. 1a). The top 20 upregulated and downregulated circRNAs were shown by hierarchical clustering (Fig. 1b).

Amongst these differentially expressed circular RNAs, we searched for those, from which the parental genes are known to function in ESCC. By browsing the human reference genome (GRCh37/hg19), we identified that circRNA_103443 is back-spliced of three exons (exon 3, 4 and 5) of Glycogen synthase kinase-3β (GSK3β) gene (chr3: 119582265–119,595,355), located at 3q22.1, and thus we named it circGSK3β. GSK3β has been reported to be a key mediator of Wnt signaling pathway which contributes to the development and progression of many malignancies, including ESCC [5, 20, 21]. However, the role of circRNA from GSK3β gene in cancer progression has not been evaluated.

### Overexpression of circGSK3β was correlates with poor patient prognosis

To investigate the clinical significance of circGSK3β in ESCC, we further collected 50 pairs of ESCC tissues and adjacent normal tissues. By using qRT-PCR, we validated that the expression of circGSK3β was frequently overexpressed in ESCC compared with that in adjacent normal tissues (Fig. 1c, Additional file 1: Figure S1A). In addition, as shown in Table 1, high circGSK3β expression levels were significantly associated with more lymph node metastasis of ESCC (Fig. 1d). To determine whether circGSK3β expression correlated with the prognosis of ESCC patients, the association of circGSK3β expression and the ESCC MFS and OS of the 50 patients were further analyzed (Fig. 1e and f, Additional file 1: Figure S1B). The median expression value of all cases was chosen as the cutoff value for separating the dataset into high and low circRNAs expression. Kaplan-Meier analysis revealed a reverse association between the expression level of circGSK3β and the MFS of the patients (Hazard ratio [HR] = 2.93; 95% CI: 1.47–6.38, *P* < 0.01), as well as with the overall survival time (Hazard ratio [HR] = 6.04; 95% CI: 1.04–10.39, *P* < 0.05).

To further characterize circGSK3β, Sanger sequencing was applied to confirm the back-splice junctions (Fig. 1g). Additionally, we designed two sets of primers: (i) divergent primers were used to amplify the circular transcripts and (ii) convergent primers were used to detect the linear transcripts, with GAPDH as a linear RNA control. The two sets of primers were then used to amplify the circular and linear transcripts of GSK3β in both cDNA and gDNA. PCR results indicated that the circular form was amplified using the divergent primers in cDNA but not gDNA, while convergent primers amplified in both cDNA and gDNA. (Fig. 1h). RNase R is an exoribonuclease that degrades linear RNAs but does not act on circular RNA. As expected, the linear transcripts of GSK3β were degraded by RNase R, whereas the circular transcripts of circGSK3β were resistant to RNase R treatment (Fig. 1I). Therefore, these data confirmed the circularity conformation of circGSK3β.

### CircGSK3β promotes ESCC cell metastasis

To evaluate the biological roles of circGSK3β in ESCC, we first constructed siRNA that could specifically silence the back-splicing region of circGSK3β (Additional file 1: Figure S2A). Depletion of circGSK3β with siRNA resulted in a significant knockdown in circGSK3β levels (Additional file 1: Figure S2B & S2D), while the expression level of the host gene, GSK3β, was not changed by either knockdown or overexpression of circGSK3β (Additional file 1: Figure S2B - S2E) in ESCC cells.

**Fig. 2:**
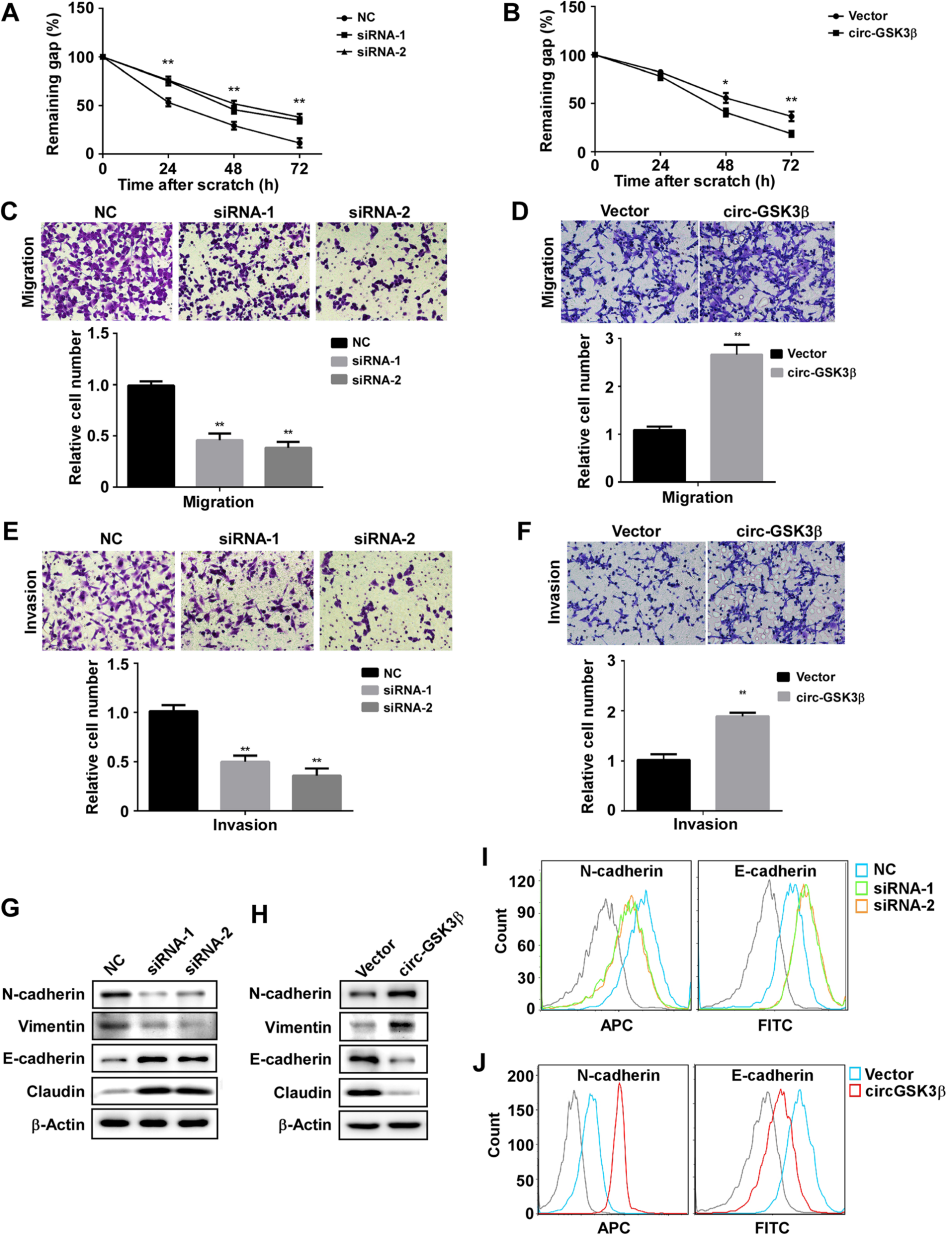
CircGSK3β promotes ESCC cell migration and invasion. **a** and **b** Scratch wound assays for circGSK3β depleted (**a**) or overexpressed (**b**) ESCC cells. The average sizes of the gaps were measured at the indicated times and expressed as mean ± S.D. of triplicates. **c** and **d** Transwell chamber assays for circGSK3β-depletion (**c**) or overexpressed (**d**) ESCC cells. The average numbers of migrated cells were counted after 24 h incubation and expressed as mean ± S.D. **e** and **f** Matrigel invasion assays for circGSK3β-depletion (**e**) or overexpressed (**f**) ESCC cells. The average numbers of migrated cells were counted after 24 h incubation and expressed as mean ± S.D. **g** and **h** EMT marker protein levels in ESCC cells with circGSK3β depletion (**g**) or overexpression (**h**) were detected by immunoblotting. **i** and **j** EMT marker protein levels in ESCC cells with circGSK3β depletion (**i**) or overexpression (**j**) were detected by Flow cytometry. *P < 0.05, **P < 0.01

To determine whether the expression level of circGSK3β affected cell motility, the scratch wound-healing assay was carried out. As shown in Fig. 2a and Fig. S3A, the scratch gap in the control cells was much smaller than that in the circGSK3β-depleted cells. In contrast, circGSK3β-overexpressed clones displayed more cells in the gap than in the control cells (Fig. 2b & Additional file 1: Figure S3B). Similarly, the Transwell assay also demonstrated that depletion of circGSK3β compromised cell migration activity (Fig. 2c), whereas circGSK3β overexpression resulted in the enhancement of transmembrane migration activity of the cells (Fig. 2d). Furthermore, the Matrigel invasion assay also showed that depletion of circGSK3β expression reduced invasion activities and overexpression of circGSK3β enhanced the invasion of ESCC cells (Fig. 2e & f). Moreover, the necessity of circGSK3β for ESCC cells migration and invasion were confirmed in another ESCC cell KYSE180 (Additional file 1: Figure S4).

It is known that epithelial-mesenchymal transition (EMT) is a critical process that induces metastasis in cancer cells [22]. Therefore, we assessed whether circGSK3β could enhance EMT of ESCC cells. Western blot analysis showed that after the knockdown of circGSK3β in ESCC cells, the expression of the epithelial marker, E-cadherin and Claudin were increased whereas the expression of the mesenchymal marker, N-cadherin and Vimentin were decreased (Fig. 2g). On the contrary, overexpression of circGSK3β led to the downregulated expression of E-cadherin and Claudin, and upregulated expression of N-cadherin and Vimentin in ESCC cells (Fig. 2h). Similarly, the Flow cytometry also demonstrated that depletion of circGSK3β compromised EMT (Fig. 2I), whereas circGSK3β overexpression resulted in the enhancement of EMT (Fig. 2j). Moreover, we confirmed the effects of circGSK3β on EMT in KYSE180 cells (Additional file 1: Figure S5). Together, these findings indicated that circGSK3β promoted ESCC cell metastasis.

### CircGSK3β promotes ESCC metastasis through GSK3β/β-catenin signaling

**Fig. 3:**
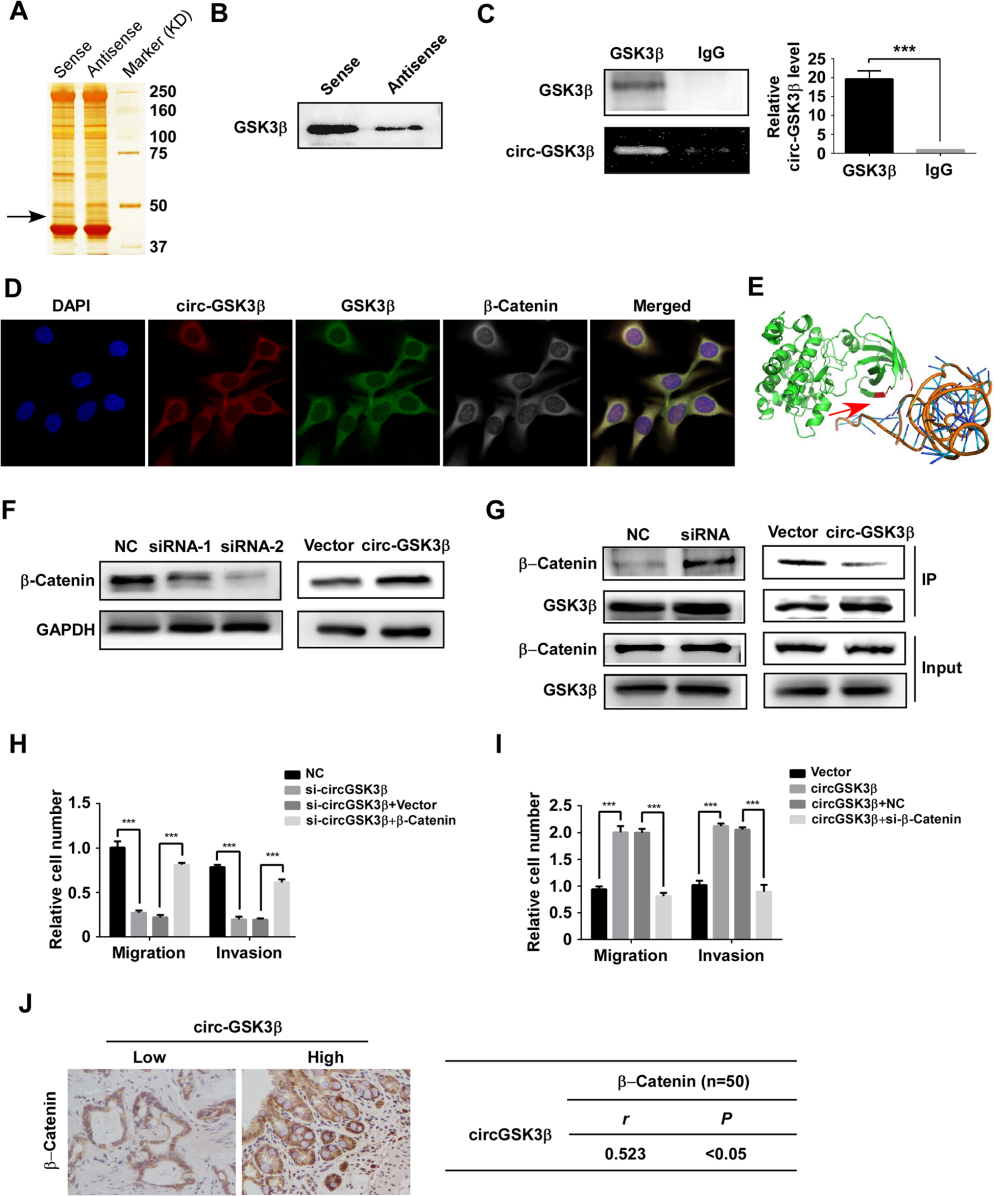
circGSK3β interacts with GSK3β and promotes metastasis by β-catenin. RNA pull-down experiment with ESCC cell lysate. The proteins were visualized by silver staining, and indicated spots were analyzed by mass spectrometry **(a)** or immunoblot of GSK3β **(b)**. **c** QPCR detection of circGSK3β retrieved by GSK3β-specific antibody compared with immunoglobulin G (IgG) in the RIP assay. **d** RNA FISH assay of circGSK3β combined with immunofluorescence detection of GSK3β and β-Catenin in ESCC cells. **e** Graphical representation of three-dimensional structures of the interaction model of circGSK3β with GSK3β by SPOT-RNA. **f** β-catenin protein levels in ESCC cells with circGSK3β depletion or overexpression were detected by immunoblotting. **g** Immunoblot detection of indicated proteins in GSK3β-immunoprecipitated complex from lysates of ESCC cells with circGSK3β knockdown or overexpression. **h** and **i** ESCC cells with depletion of circGSK3β (Η) were transfected with β-catenin and circGSK3β-overexpressed cells (**i**) were transfected with control siRNA or siRNA against β-catenin. Migration and invasion abilities of ESCC cells were detected by Transwell and Matrigel invasion assay, respectively. **j** Statistical analyses of the association between circGSK3β and β-catenin expression in ESCC tissues. *r*, Pearson correlation coefficient; *P < 0.05, **P < 0.01, ***P < 0.001

Most mechanisms reported that the mechanism-of-action of circRNA was the sequestration of microRNA to restore mRNA translation as a competing endogenous RNA [23, 24]. However, RNA-binding protein Immunoprecipitation (RIP) revealed that circGSK3β was not associated with the Argonaute-2 (AGO2) protein, a key component of the microRNA-containing RISC complex [25]. Therefore, circGSK3β might exert its function in a different manner. To elucidate the downstream target of circGSK3β that contributes to ESCC progression, we next performed pull-down assays with biotinylated circGSK3β, followed by mass spectrometry to search for potential circGSK3β-interacting proteins. Interestingly, a major differential band precipitated in ESCC cell lysates was identified to be GSK3β, the parental protein of circGSK3β (Fig. 3a). The interaction between circGSK3β and GSK3β was further validated through pull-down by circGSK3β (Fig. 3b) and RIP analysis (Fig. 3c). Consistently, RNA fluorescent in situ hybridization (FISH) followed by immunofluorescence (IF) also demonstrated that circGSK3β and GSK3β were predominantly colocalized in the cytoplasm (Fig. 3d). To explore the potential binding domain on GSK3β for circGSK3β, computational simulation was performed using SPOT-RNA (http://sparks-lab.org/yueyang/server/SPOT-Struct-RNA/) [26]. The structure of GSK3β protein used in the procedure was derived from Protein Data Bank (PDB) entry 1h8f. The predicted complex structure of GSK3β and circGSK3β was shown in Fig. 3e and the binding residues labeled in red are located at the N-terminal of GSK3β that is important for its activity [27].

As a pro-oncogenic factor, β-catenin is one of the most important regulators that trigger the EMT of ESCC. It’s known that β-catenin is phosphorylated by GSK3β and sequentially ubiquitinated and targeted for degradation [21, 28]. We next investigated whether the activity of β-catenin was regulated by circGSK3β. Western blot showed that the abundance of β-catenin was reduced in circGSK3β-depleted cells, while increased in circGSK3β overexpressing ESCC cells (Fig. 3f). Of note, neither depletion nor overexpression of circGSK3β affected the expression of β-catenin at the mRNA level (Additional file 1: Figure S6). Since the activity of β-catenin is mainly regulated through GSK3β induced phosphorylation, we hypothesized that circGSK3β activate β-catenin by interacting with GSK3β to prevent phosphorylation of β-catenin by GSK3β. Coimmunoprecipitation confirmed that knockdown of circGSK3β promoted the association of GSK3β with β-catenin, and overexpression of circGSK3β attenuated GSK3β and β-catenin interaction in ESCC cells (Fig. 3g). These results demonstrated that circGSK3β could inhibit the activity of GSK3β by interacting with it.

To investigate whether β-catenin was required for promotion of metastasis by circGSK3β in ESCC cells, β-catenin was overexpressed in ESCC cells with circGSK3β depletion or depleted in ESCC cells with circGSK3β overexpression. The Transwell and Matrigel invasion assay showed that overexpression of β-catenin compensated the loss of migration and invasion due to depletion of circGSK3β (Fig. 3h & Additional file 1: Figure S7A). On the other hand, depletion of β-catenin attenuated the increase of migration and invasion ability caused by circGSK3β overexpression (Fig. 3i & Additional file 1: Figure S7B). To further prove that β-catenin activation by up-regulation of circGSK3β is critical for ESCC tumorigenesis, we examined the correlation between circGSK3β and β-catenin activation in ESCC tissues. IHC was performed to examine β-catenin expression in ESCC specimens. A significant positive correlation between β-catenin and circGSK3β expression was observed (Pearson contingency coefficient: *r* = 0.523, *P*< 0.05; Fig. 3j). Taken together, our results demonstrate that circGSK3β drives metastasis in ESCC through an altered GSK3β/β-catenin pathway.

### Ablation of circGSK3β inhibited the tumorigenicity of ESCC cells

**Fig. 4:**
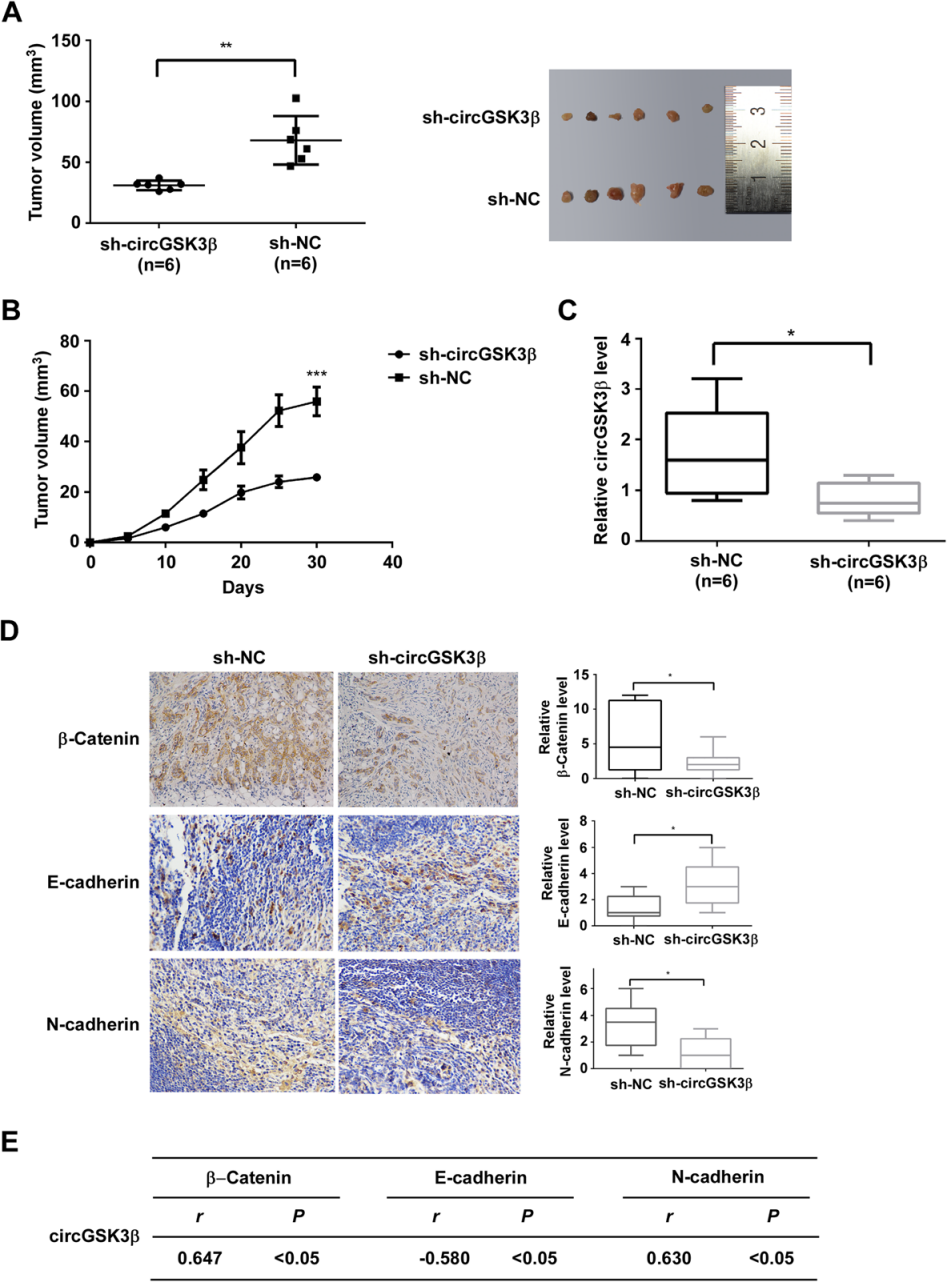
Ablation of circGSK3β inhibits the tumorigenicity of ESCC cells. **a** and **b** ESCC cells with or without depletion of circGSK3β were grafted to the flank of nude mice by injecting the cells subcutaneously to observe tumor development, and volumes of tumor were determined and plotted as mean ± S.D. of six independent experiments. **c** The mRNA levels of circGSK3β in transplanted tumor tissues were determined by qRT-PCR. **d** Expression levels of β-catenin, E-cadherin and N-cadherin in tumors were determined by immunohistochemistry analysis. **e** Statistical analyses of the association between circGSK3β and β-catenin, E-cadherin or N-cadherin in transplanted tumor tissues. *r*, Pearson correlation coefficient. *P < 0.05. **P < 0.01, ***P < 0.001

To further investigate whether circGSK3β contributed to tumor progression in vivo, ESCC cells with or without circGSK3β depletion were grafted to the flank of nude mice. The tumor sizes were measured with a Vernier caliper and the tumors were excised for analyses 30 days after implantation. As shown in Fig. 4a, tumors derived from circGSK3β-depleted cells were smaller and had a reduced growth rate compared with the control (Fig. 4b).

Next, circGSK3β expression of the tumors was detected by qRT-PCR (Fig. 4c) and the abundance of β-catenin, E-cadherin, and N-cadherin were assessed by immunohistochemical staining. Statistical analyses of expression of these proteins were shown in Fig. 4d. The results have consistently demonstrated that expression of β-catenin and N-cadherin were lower in circGSK3β-deficient tumors than that in control tumors, and there is a negative correlation between circGSK3β expression and E-cadherin (Fig. 4e).

### Plasma circGSK3β is a potential biomarker for ESCC diagnosis and prognosis

**Fig. 5:**
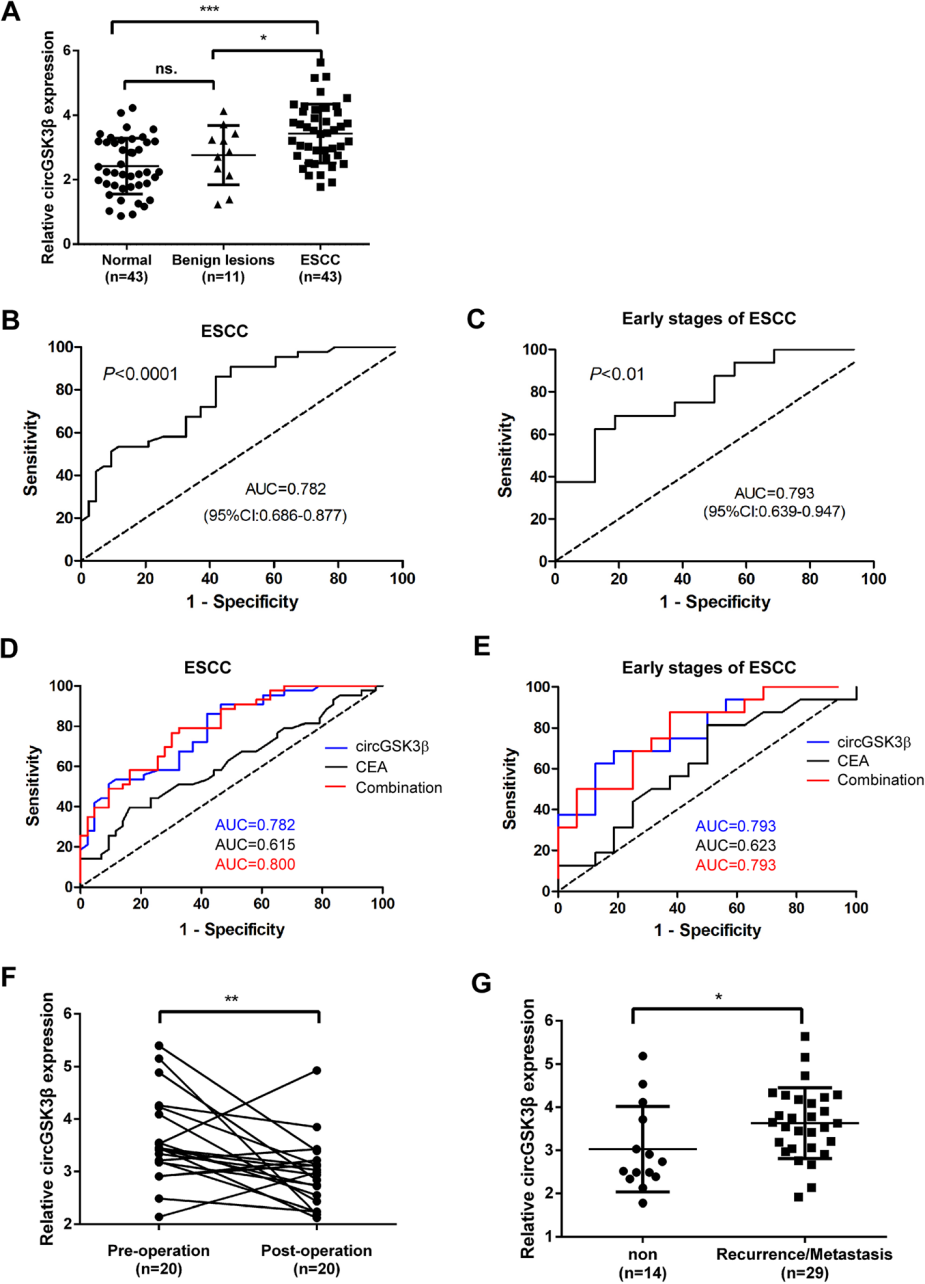
Plasma level of circGSK3β is a potential biomarker for ESCC. **a** The relative levels of circGSK3β in patients with healthy controls, benign lesions, and ESCC. **b** and **c** ROC curves showing plasma levels of circGSK3β in ESCC patients (**b**) and early stages of ESCC patients (**c**). **d** and **e** The comparison of ROC curves of circGSK3β, CEA and the combination of circGSK3β, CEA. **f** The pre-operative plasma level of circGSK3β and post-operation. **g** The comparison of percentage of patients with metastasis 10 months after the surgery in high vs. low circGSK3β level. ns., not significant. * *P* < 0.05; ** *P* < 0.01; and *** *P* < 0.001

Since circRNAs have a circular covalently-bonded structure, they have higher inherent tolerance to exonuclease digestion and prolonged lifetime in systemic circulation [29]. The stability and easy detectability make circRNAs an ideal candidate to serve as “liquid biopsy” biomarkers for cancer detection. To determine whether the plasma level of circRNA can serve as a biomarker for ESCC diagnosis and prognosis, the abundance of circGSK3β in the plasma in all groups (ESCC patients, patients with benign lesion and normal control) were quantified via ddPCR. As shown in Fig. 5a, compared with healthy controls and benign lesions, the abundance of circGSK3β in the plasma was significantly increased in ESCC patients.

Early diagnosis and treatment of ESCC is of great values to improve the OS of ESCC patients. To determine whether the plasma level of circGSK3β had ESCC diagnostic value, ROC curve was applied to analyze the diagnostic sensitivity and specificity, and Youden Index was used to select the optimal cutoff. The area under curve (AUC) for circGSK3β in distinguishing ESCC from normal controls was 0.782 (95% CI: 0.686–0.877) (Fig. 5b). At the threshold of 0.442, the optimal sensitivity and specificity of circGSK3β were 86.05 and 58.14%, respectively. We next determined whether the plasma level of circGSK3β could be used for early detection of ESCC (stage I + stage II). According to the ROC curve, the AUC for circGSK3β in ESCC at early stages and healthy controls reached 0.793 (95% CI: 0.639–0.947) (Fig. 5c), with a sensitivity of 68.75% and specificity of 81.25%. Furthermore, we validated the biomarker potential of circGSK3β in an independent validation cohort comprising of 43 patients with ESCC and 43 healthy controls (Additional file 1: Figure S8). The AUC of circGSK3β for ESCC or early stages of ESCC and controls in the validation cohort were 0.8012 (95% CI: 0.7075–0.8950) and 0.8255 (95% CI: 0.6929–0.9581), respectively. These findings validate the performance of circGSK3β as a plasma marker for the detection of ESCC and early stages of ESCC.

Currently, CEA is one of the most commonly used diagnosis markers for ESCC [15]. Therefore, the performance of circGSK3β and CEA in detecting ESCC and early stages of ESCC was compared. As shown in Fig. 5d, the AUC of CEA was low to 0.615. CEA also showed lower sensitivity than that of circGSK3β, although the specificity was higher (Additional file 1: Figure S9). We next examined whether circGSK3β could be complementary to the use of CEA for ESCC diagnosis. By combining with circGSK3β and CEA, the AUC reached 0.800 and the specificity was increased to 67.4%, but the sensitivity was dropped to 79.1%. Consistently, the combined analyses of circGSK3β and CEA in early stages of ESCC dramatically increased the diagnostic sensitivity (87.5%) (Fig. 5e & Additional file 1: Figure S9). Taken together, these results indicated that the combinatory use of circGSK3β with CEA may provide a new promising biomarker for early diagnosis of ESCC.

To estimate whether the plasma levels of circGSK3β had predictive values for clinical improvement after surgery, the plasma levels of circGSK3β in 20 patients were compared before and after surgery. Data showed that the plasma levels of circGSK3β in 15 (15/20 = 75%) patients were reduced after surgery (Fig. 5f). To determine whether plasma circGSK3β can predict recurrence/metastasis in patients with ESCC, the circGSK3β expression and patient history from 43 ESCC patients were analyzed. The results showed that the levels of circGSK3β in patients with recurrence/metastasis 10 months after surgery (*n* = 29) was much higher than in patients without recurrence/metastasis (*n* = 14; Fig. 5g), suggesting that plasma circGSK3β level may be a valuable predictor of ESCC recurrence/ metastasis.

## Discussion

Although the biogenesis and expression of circRNAs have been intensively investigated in the recent years, the functions of circRNAs in cancers are still not well understood. In this study, we found that the expression of several circRNAs is enriched in ESCC. Among them, circGSK3β, a circRNA derived from GSK3β, was upregulated in all stages of ESCC. Overexpression of circGSK3β potentiates multiple tumor characteristics, including migration and invasion. These functions greatly contribute to cancer metastasis and malignancy, which supports the observation that high levels of circGSK3β are associated with poor prognosis of ESCC patients. Although several oncogenes have been identified as playing critical roles in the development of ESCC, whether noncoding RNA, especially circRNAs, play a role in ESCC metastasis is largely unknown. To our knowledge, this is the first report that thoroughly investigates the expression, function, and clinical implication of circRNA derived from GSK3β gene in ESCC.

Studies from mouse and human cell lines have shown that many orthologous circRNAs are evolutionarily conserved, suggesting specific roles of circRNAs in cellular physiology. Many researchers have found that some circRNAs contain miRNA binding sites and may function as sponges to arrest miRNA functions. Some circular RNAs may interact with RNA-binding proteins which form RNA–protein complex and regulate their activity. However, the mechanism by which circRNAs regulates their parental proteins remains unknown. In this study, we found that circGSK3β could inhibit the biogenesis of its parental protein, GSK3β, which functions as a tumor suppressor by inhibiting the Wnt/β-catenin pathway-mediated cell metastasis. Different from most previously identified suppressors, circGSK3β operates at the level of β-catenin by directly interacting with and inhibiting GSK3β-induced phosphorylation, which represents a different layer of negative regulation on GSK3β/β-Catenin pathway. Our work suggests that circRNA can affect cellular metastasis and function by directly interacting with signaling molecules and regulating their signaling transduction, providing a novel mechanism of circRNA in cancer progression. GSK3β/β-Catenin signaling is critical for tumor growth, maintenance, and metastasis. Many studies have demonstrated that β-Catenin inhibitors have a significant therapeutic value [30–32]. In this regard, circGSK3β can be a potential druggable target for cancer treatment. Our result indicated that ablation of circGSK3β inhibits tumor growth which is the direct evidence that the inhibition of circGSK3β has the potential for ESCC treatments. Furthermore, our result shows that high expression of circGSK3β correlates with disease aggressiveness and poor outcomes of ESCC patients, including short MFS and OS.

Clinical data shows that the 5-year survival rate of early stage of ESCC patients after surgery is much higher than that of ESCC [33]. However, due to no obvious symptoms of early ESCC and the lack of sensitive detection methods for early diagnosis, the majority of ESCC patients are diagnosed at the advanced stages and lost the best therapeutic window for treatment. Currently, traditional tumor markers, such as CEA and cytokeratin 19 fragment (Cyfra) 21–1, are used to diagnose and evaluate ESCC progression. However, the sensitivity and validity of CEA and Cyfra 21–1 detection are insufficient for early ESCC detection [15, 34]. Therefore, improved biomarkers that allow early ESCC detection are urgently needed. CircRNAs are recently identified as noncoding RNAs that are highly prevalent in eukaryotic transcriptome. A growing number of studies have demonstrated that circRNAs play a significant role in the pathogenesis of cancers [12, 13, 35–37]. The intrinsic circular characteristics render circRNAs unusually stable both inside cells and in extracellular plasma [9]. Therefore, compared to traditional biopsy biomarkers in tumor tissues, circRNAs show great potential values as tumor markers for cancer diagnosis. However, the diagnostic values of peripheral blood circRNAs for ESCC remain vague. Our previous study identified that the plasma level of long noncoding RNA (lncRNA) HOTAIR was significantly higher in ESCC patients compared with that of healthy controls and might serve as a potential biomarker for ESCC [38]. In this study, we clearly demonstrated that the AUC for circGSK3β was much higher than CEA in the diagnosis of ESCC or early stages of ESCC. Furthermore, the use of plasma circGSK3β in combination with CEA could be used as effective plasma biomarkers for ESCC. A comprehensive large cohort study in ESCC are needed to validate these findings.

## Conclusions

In summary, our findings report that circGSK3β is an upstream regulator for GSK3β/β-Catenin signaling, which interacts with GSK3β to promote the activity of β-catenin in ESCC cells. The overexpression of circGSK3β is associated with metastasis and poor prognosis in ESCC patients. Furthermore, the in vivo experiments and clinical characteristics of circGSK3β indicate that suppression of circGSK3β can be have indicative therapeutic value for ESCC and that the expression level of plasma circGSK3β has the potential to serve as a biomarker for ESCC diagnosis and prognosis.

## Additional file

**Additional file 1: Figure S1.** Expression of circGSK3β in human ESCC. **Figure S2.** Depletion and overexpression of circGSK3β in ESCC cells. **Figure S3.** circGSK3β promoted TE1 cell motility. **Figure S4.** CircGSK3β promotes KYSE180 cell migration and invasion. **Figure S5.** CircGSK3β promotes KYSE180 cell EMT. **Figure S6.** circGSK3β does not affect mRNA levels of β-catenin. **Figure S7**. CircGSK3β promotes ESCC migration and invasion through β-catenin. **Figure S8**. The diagnosis value between ESCC or early stages of ESCC and normal controls in the validation cohort. **Figure S9**. The diagnosis value between ESCC or early stages of ESCC and normal controls.

## Data Availability

The primary data in the microarray analysis have been uploaded to the Gene Expression Omnibus with the accession number GSE131969. The rest of datasets used and analyzed during the current study are available from the corresponding author on reasonable request.

## Abbreviations

AUC: Area under curve
CEA: Carcinoembryonic antigen
circRNAs: Circular RNAs
ddPCR: Droplet digital PCR
EMT: Epithelial-mesenchymal transition
ESCC: Esophageal squamous cell carcinoma
FISH: Fluorescence in situ hybridization
HR: Hazard ratio
IB: Immunoblotting
IP: Immunoprecipitation
lncRNA: long noncoding RNA
MFS: Metastasis-free survival
miRNA: microrna
OS: Overall survival
RIP: RNA-binding protein Immunoprecipitation
ROC: Receiver operating characteristic
shRNA: Short hairpin RNA
