# Supplementary material for: circGSK3β promotes metastasis in esophageal squamous cell carcinoma by augmenting β-catenin signaling
Source: Mol Cancer. 2019 Nov 14;18:160. doi: 10.1186/s12943-019-1095-y (PMC6854808; doi:10.1186/s12943-019-1095-y)
Supplement: Supplementary file 1 — Additional file 1: Figure S1. Expression of circGSK3β in human ESCC. Figure S2. Depletion and overexpression of circGSK3β in ESCC cells. Figure S3. circGSK3β promoted TE1 cell motility. Figure S4. CircGSK3β promotes KYSE180 cell migration and invasion. Figure S5. CircGSK3β promotes KYSE180 cell EMT. Figure S6. circGSK3β does not affect mRNA levels of β-catenin. Figure S7. CircGSK3β promotes ESCC migration and invasion through β-catenin. Figure S8. The diagnosis value between ESCC or early stages of ESCC and normal controls in the validation cohort. Figure S9. The diagnosis value between ESCC or early stages of ESCC and normal controls. [file 12943_2019_1095_MOESM1_ESM.pdf]

A

	T>N	T=N	T<N
circGSK3β	70% (35/50)	0 (0/50)	30% (15/50)

B

	MFS				OS			
	n	HR	95%CI	*P*	n	HR	95%CI	*P*
circGSK3β								
Low	9	1			14	1		
High	11	2.931	1.468-6.379	<0.01	23	6.035	1.038-10.39	<0.05

Figure S1. Expression of circGSK3β in human ESCC. (A) Statistical comparison of differences in expression of circGSK3β in human ESCC and adjacent non-tumor tissues. (B) Statistical analyses of the association of circGSK3β expression with overall survival time and metastasis free survival time of ESCC patients. *n*: sample number; OS: overall survival; MFS: Metastasis-free survival; CI: confidence interval; HR: hazard ratio; *P*: P-value.

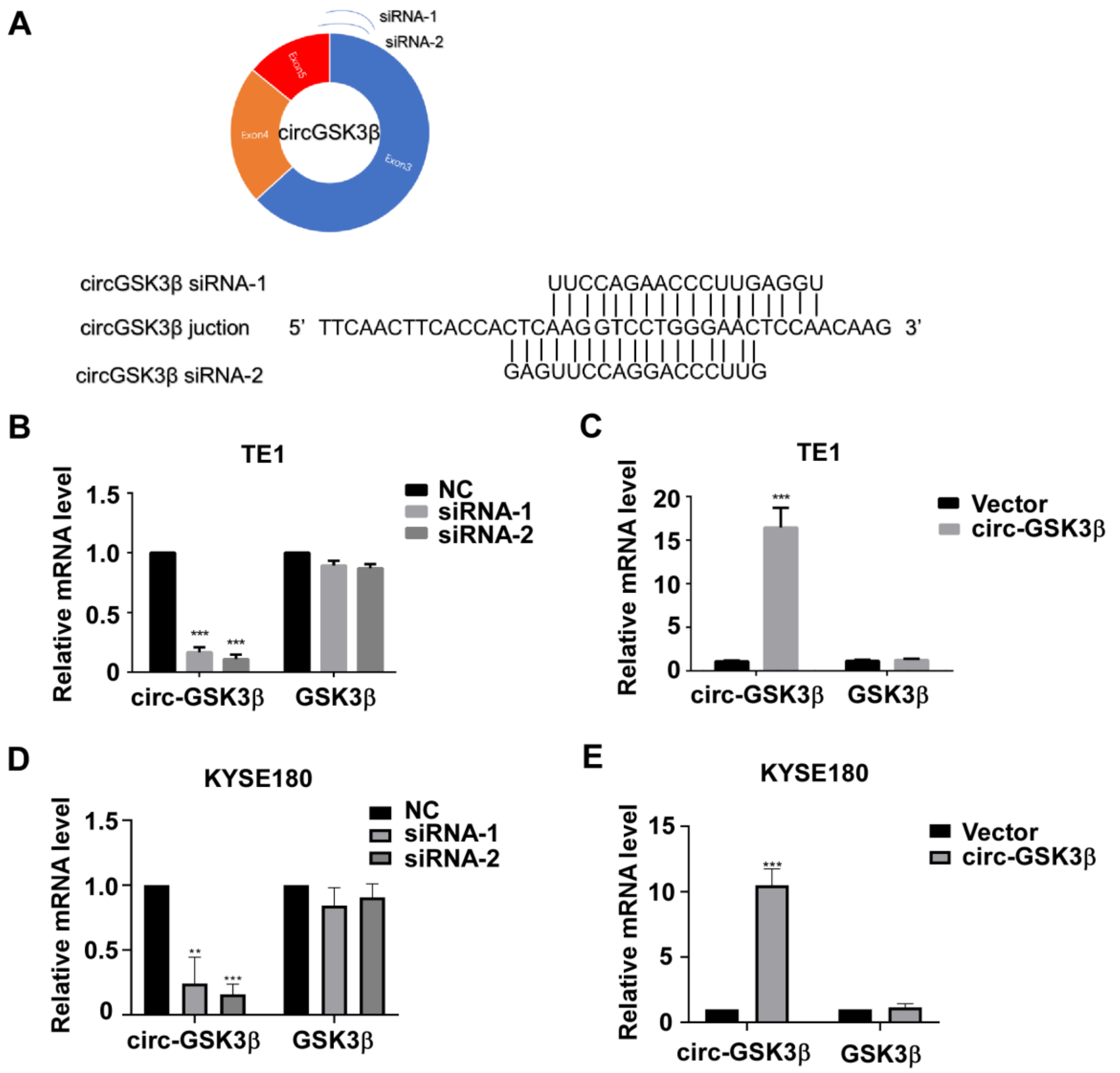

Figure S2. Depletion and overexpression of circGSK3β in ESCC cells. (A) Schematic illustration showed two siRNAs targeting circGSK3β. si-circGSK3β targets the back-splice junction of circGSK3β. **(B&C)** qRT-PCR for circGSK3β and GSK3β mRNA levels in TE1 cells with depletion (B) or overexpression (C) of circGSK3β. **(D&E)** qRT-PCR for circGSK3β and GSK3β mRNA levels in KYSE180 cells with depletion (D) or overexpression (E) of circGSK3β. Data are represented as the mean ± S.D. of triplicates. $^{**}P < 0.01$; $^{***}P < 0.001$.

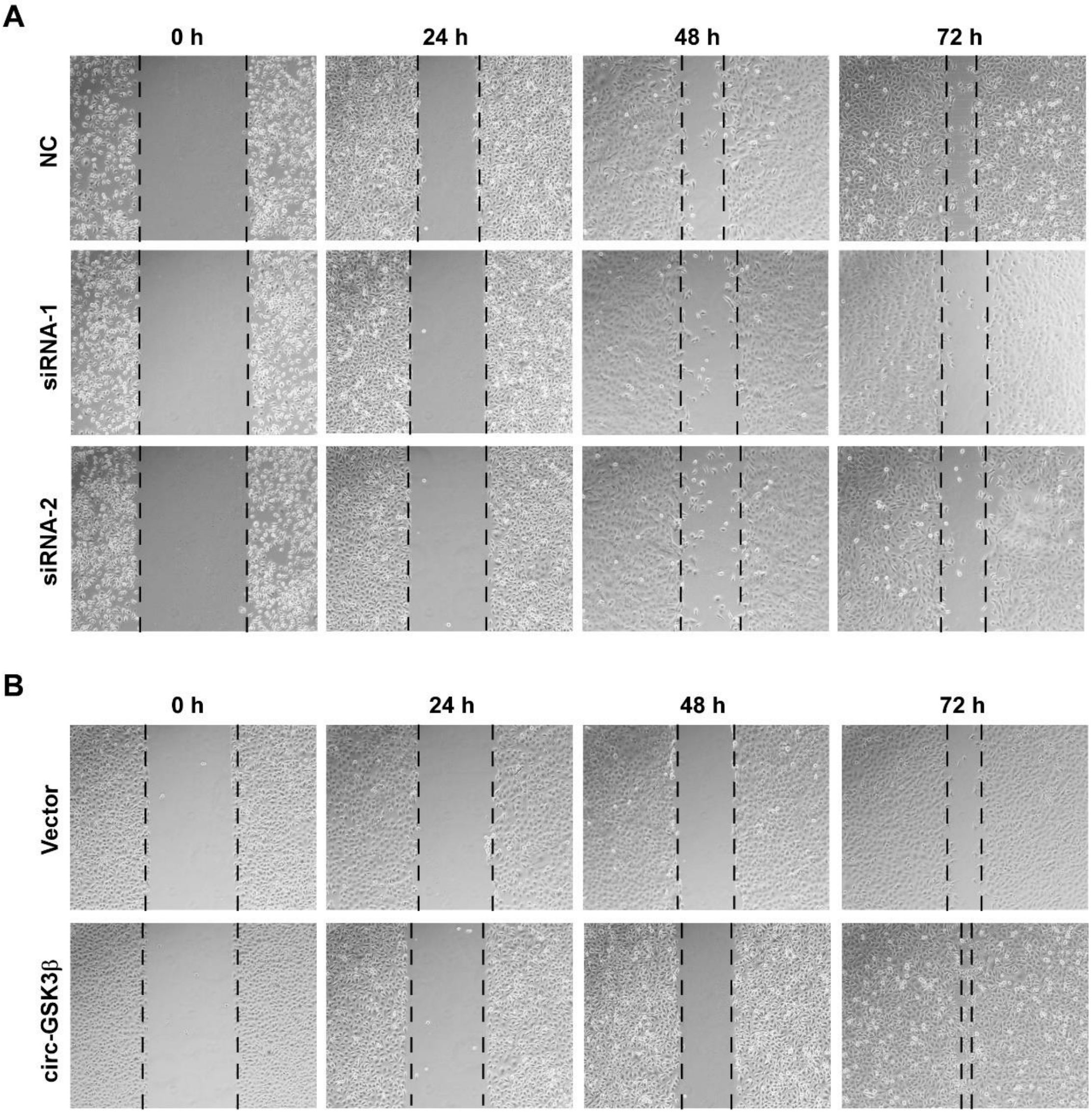

Figure S3. circGSK3β promoted TE1 cell motility. The cell motility was analyzed by the scratch wound healing assays as described in Methods. Representative images were shown.

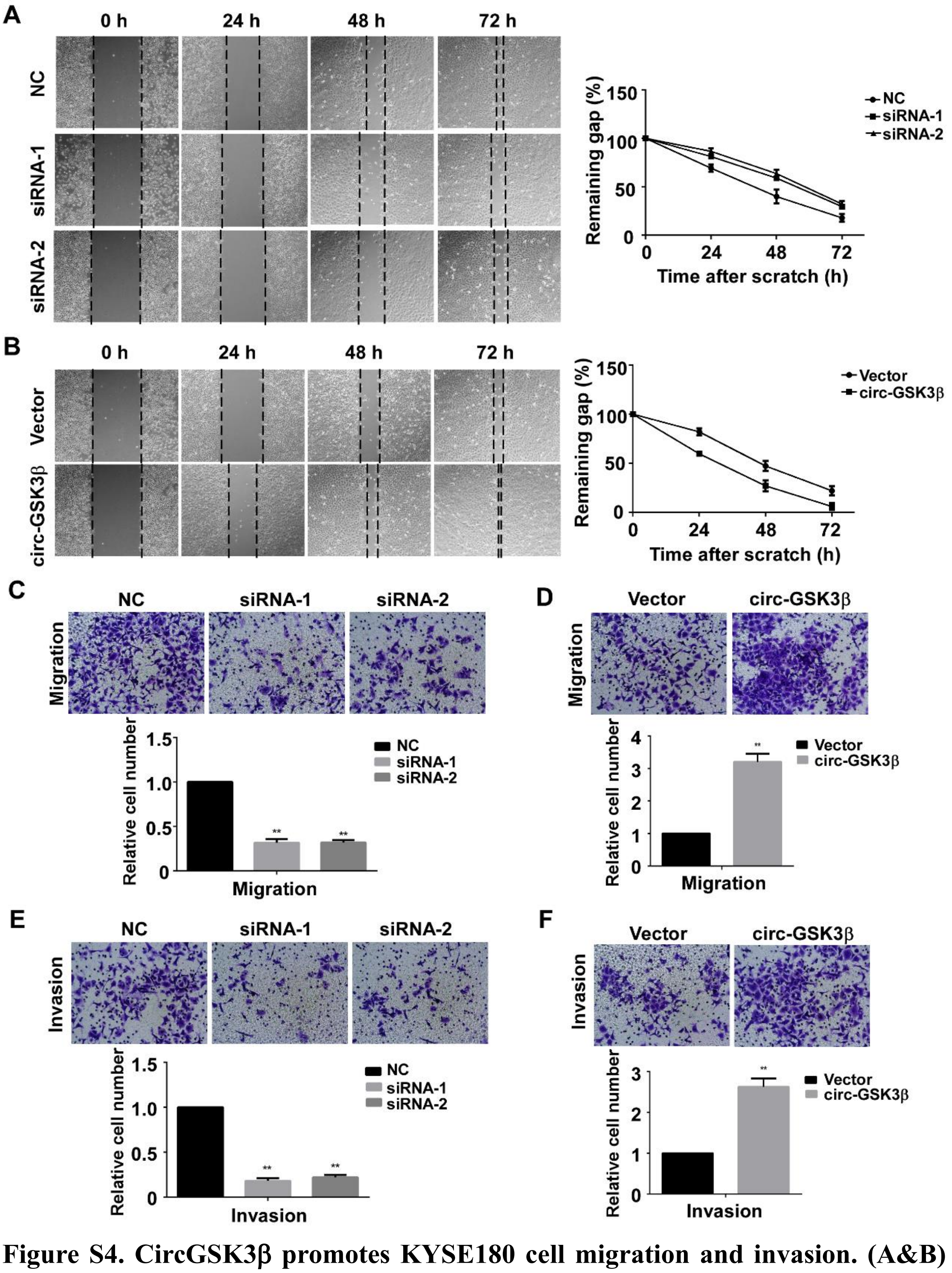

Figure S4. CircGSK3β promotes KYSE180 cell migration and invasion. (A&B) Scratch wound assays for circGSK3β depleted (A) or overexpressed (B) KYSE180 cells. The average sizes of the gaps were measured at the indicated times and expressed as mean ± SD of triplicated samples. **(C&D)** Transwell chamber assays for circGSK3β-depletion (C) or overexpressed (D) KYSE180 cells. The average numbers of migrated cells were counted after 24 h incubation and expressed as mean ± S.D. **(E&F)** Matrigel invasion assays for circGSK3β-depletion (E) or overexpressed (F) KYSE180 cells. The average numbers of migrated cells were counted after 24 h incubation and expressed as mean ± S.D. $^{**}P < 0.01$.

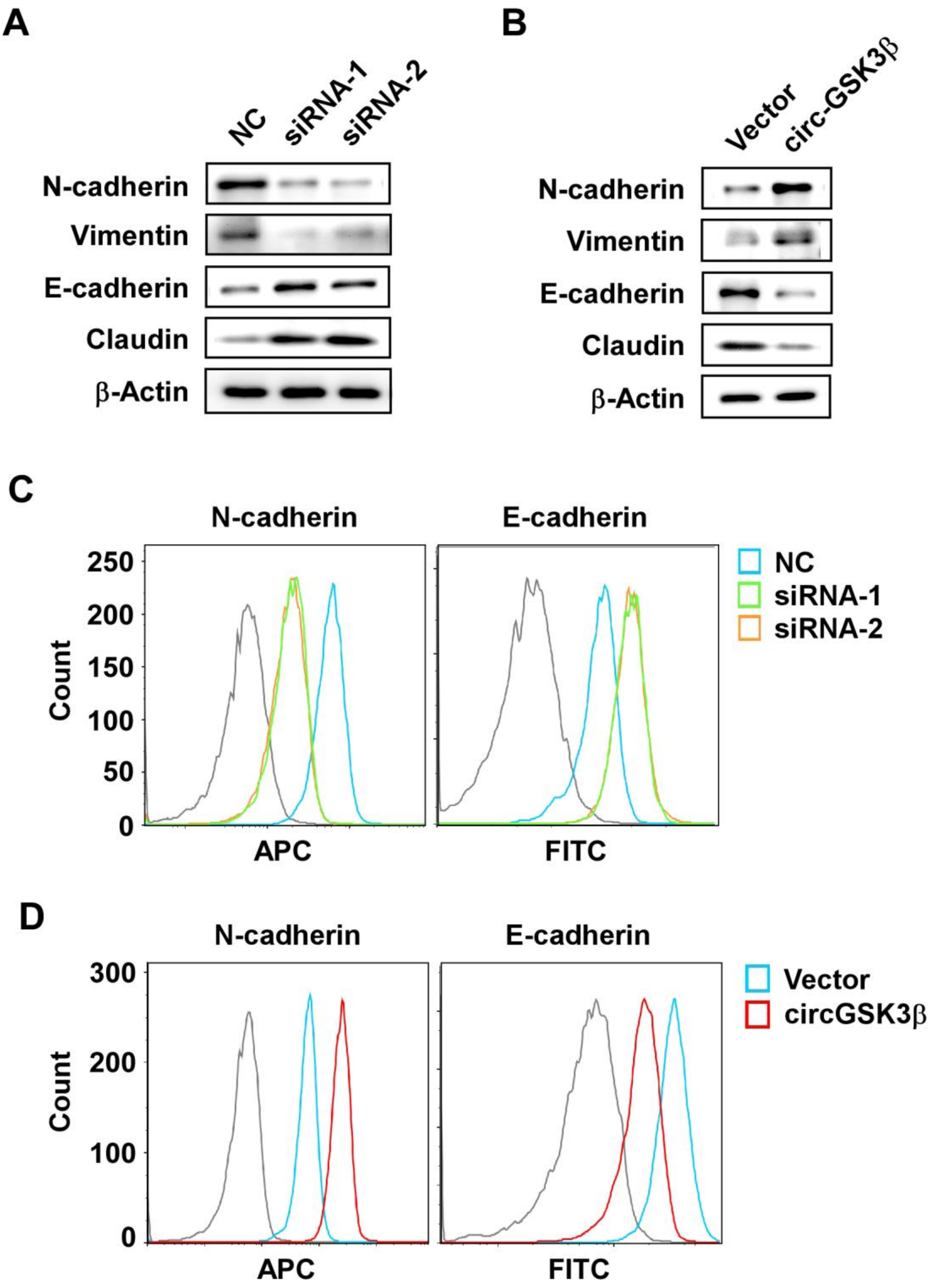

Figure S5. CircGSK3β promotes KYSE180 cell EMT. (A&B) EMT marker protein levels in KYSE180 cells with circGSK3β depletion (A) or overexpression (B) were detected by immunoblotting. **(C&D)** E-cadherin and N-cadherin protein levels in KYSE180 cells with circGSK3β depletion (C) or overexpression (D) were detected by Flow cytometry.

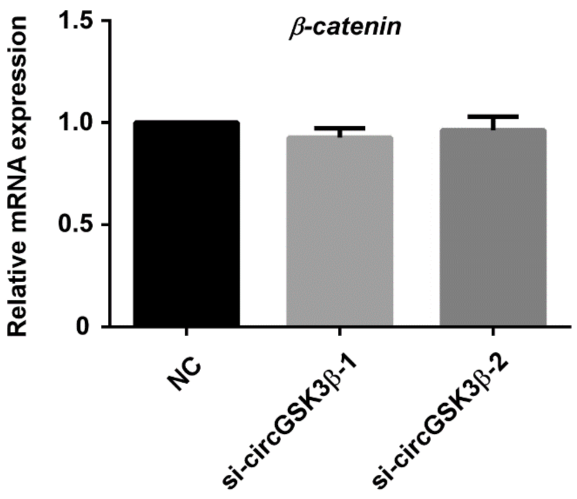

Figure S6. circGSK3β does not affect mRNA levels of β-catenin. TE1 cells with depletion of circGSK3β and the control cells were harvested and the β-catenin mRNA levels were determined by real-time RT-PCR assay. NC: negative control.

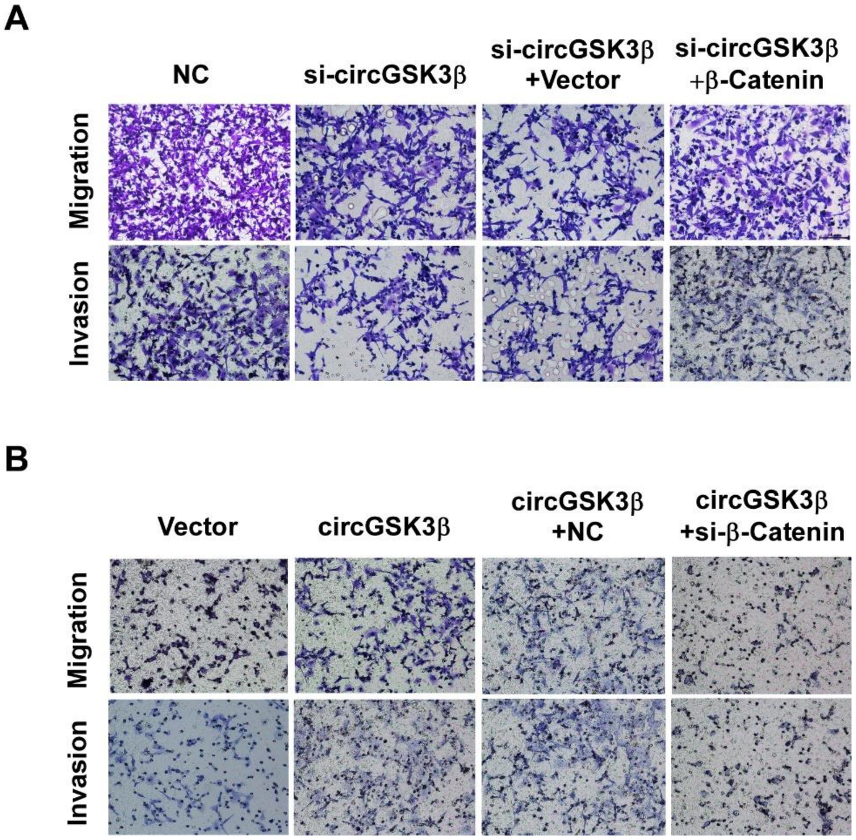

Figure S7. **CircGSK3β promotes ESCC migration and invasion through β-catenin**. ESCC cells with depletion of circGSK3β were transfected with β-catenin **(A)** and circGSK3β-overexpressed cells were transfected with control siRNA or siRNA against β-catenin **(B)**. Migration and invasion abilities of ESCC cells were detected by Transwell and Matrigel invasion assay, respectively.

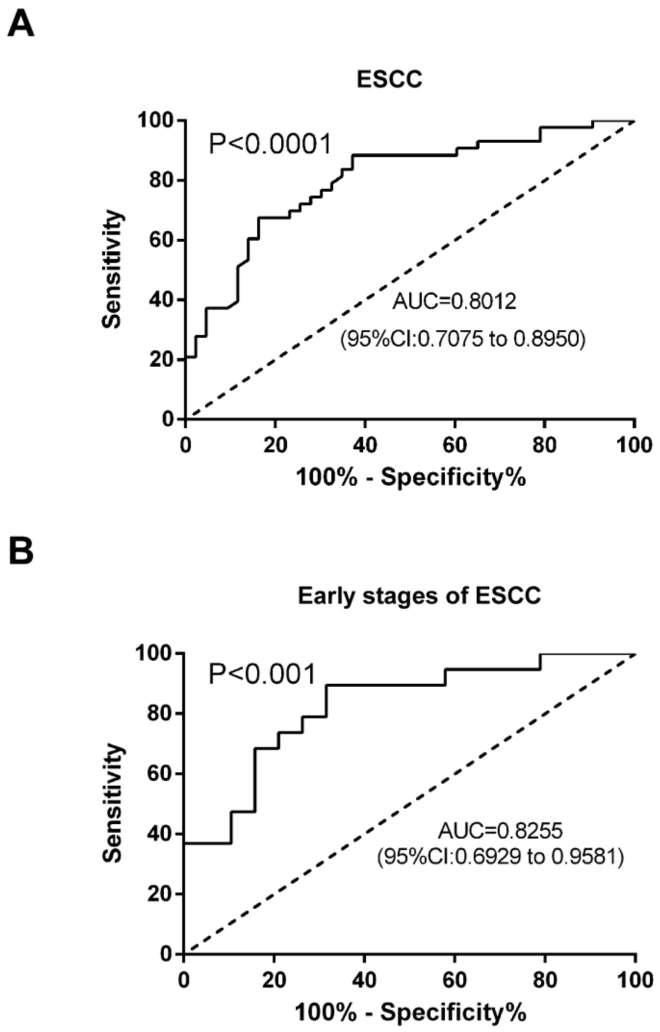

Figure S8. **The diagnosis value between ESCC or early stages of ESCC and normal controls in the validation cohort**. ROC curves showing plasma levels of circGSK3β in ESCC patients (A) and early stages of ESCC patients (B).

Variables	ESCC			Early stage of ESCC		
	circGSK3β	CEA	Combination	circGSK3β	CEA	Combination
ROC	0.782	0.615	0.800	0.793	0.623	0.793
Sensitivy	86.05%	39.53%	79.10%	68.75%	81.25%	87.50%
Specificity	58.14%	83.72%	67.40%	81.25%	50%	62.50%

Figure S9. **The diagnosis value between ESCC or early stages of ESCC and normal controls**. ROC curves were applied to analysis the diagnostic values of circRNA and CEA. Youden index (sensitivity + specificity-1) was chosen to identify the optimal cut-off threshold values.
